# Assessing Cumulative Health Risks from Exposure to Environmental Mixtures—Three Fundamental Questions

**DOI:** 10.1289/ehp.9333

**Published:** 2007-01-24

**Authors:** Ken Sexton, Dale Hattis

**Affiliations:** 1 University of Texas School of Public Health, Brownsville Regional Campus, Brownsville, Texas, USA; 2 The George Perkins Marsh Institute, Clark University, Worcester, Massachusetts, USA

**Keywords:** chemical mixtures, combined effects, cumulative exposure, cumulative risk, environmental mixtures, interaction mechanisms, multiple stressors, risk ssessment

## Abstract

Differential exposure to mixtures of environmental agents, including biological, chemical, physical, and psychosocial stressors, can contribute to increased vulnerability of human populations and ecologic systems. Cumulative risk assessment is a tool for organizing and analyzing information to evaluate the probability and seriousness of harmful effects caused by either simultaneous and/or sequential exposure to multiple environmental stressors. In this article we focus on elucidating key challenges that must be addressed to determine whether and to what degree differential exposure to environmental mixtures contributes to increased vulnerability of exposed populations. In particular, the emphasis is on examining three fundamental and interrelated questions that must be addressed as part of the process to assess cumulative risk: *a*) Which mixtures are most important from a public health perspective? and *b*) What is the nature (i.e., duration, frequency, timing) and magnitude (i.e., exposure concentration and dose) of relevant cumulative exposures for the population of interest? *c*) What is the mechanism (e.g., toxicokinetic or toxicodynamic) and consequence (e.g., additive, less than additive, more than additive) of the mixture’s interactive effects on exposed populations? The focus is primarily on human health effects from chemical mixtures, and the goal is to reinforce the need for improved assessment of cumulative exposure and better understanding of the biological mechanisms that determine toxicologic interactions among mixture constituents.

It is well established that people are exposed to a diverse and dynamic mixture of environmental stressors as a routine part of their existence, and there is clear evidence that toxicity can be modified by simultaneous or sequential exposure to multiple environmental agents (Carpenter et al. 2002; Hertzberg and Teuschler 2002). We know, for example, that exposure to tobacco smoke and asbestos (Erren et al. 1999) or radon (Morrison et al. 1998) multiplicatively increases the risk of lung cancer over what would be expected from simple addition of the effects from the agents acting separately. Similarly, exposure to aflatoxin-contaminated food and hepatitis B infection greatly increases the risk of hepato-cellular carcinoma (Kuper et al. 2001), exposure to noise and toluene results in higher risk of hearing loss than from either stressor alone (Franks and Thais 1996), exposure to poly-cyclic aromatic hydrocarbons and ultraviolet radiation increases toxicity to aquatic organisms (Oris and Geisy 1985), and adults with increased perceived stress (Cohen et al. 1999) and children of parents experiencing stress (Boyce et al. 1995) are more susceptible to viral respiratory infections.

Risk assessments have, nevertheless, focused mainly on the narrow question of harm from exposure to individual chemicals in a specific environmental medium via a single route or pathway [U.S. Environmental Protection Agency (EPA) 2003]. Although there is an expanding body of work on cumulative exposures and combined effects on people (Agency for Toxic Substances and Disease Registry 2002; Carpenter et al. 2002; Monosson 2005; U.S. EPA 2000; Yang 1994) and on ecosystems (Bryce et al. 1999; Dale et al. 1998; U.S. EPA 1998), adequate and appropriate data are rarely available to conduct a rigorous assessment of cumulatve risk. In this article, we briefly review three fundamental and interrelated questions that must be addressed as part of the cumulative risk assessment process. Which environmental mixtures are most important from a public health perspective? What is the nature and magnitude of cumulative exposures for populations of interest? What is the mechanism and consequence of combined effects on exposed populations?

## Which Environmental Mixtures Are Most Important?

For our purposes, the terms “agent” and “stres-sor” are used interchangeably to mean any biological (e.g., *Staphylococcus aureus*, *Penicillium funiculosum*), chemical [e.g., benzene, lead, dichlorodiphenyltrichloroethane (DDT)], or physical (e.g., heat, noise, radiation) entity, or psychosocial demand or challenge (e.g., family conflict, unemployment, neighborhood crime) that can, either by its presence or absence, cause deleterious effects in an organism, community, or population (U.S. EPA 2003). A mixture is defined as a combination of two or more environmental agents.

### Types of mixtures

Faced with the array, complexity, and variability of real-world mixtures, scientists have found it useful to distinguish among three generic types: similar, defined, and coincidental mixtures (Sexton et al. 1995a; Vouk et al. 1987). Similar mixtures (see Supplemental Material, Appendix 1, Table 1-1; http://www.ehponline.org/docs/2007/9333/suppl.pdf) are composed of agents that have comparable properties, such as chemical structure, mechanism of toxic action, or toxicologic end point (e.g., organophosphate pesticides). Similar mixtures, especially those with a common mode of toxicity, and for which potency of all can be summarized in terms of dosage of one specific reference chemical in the group, are the most amenable to quantitative assessment of cumulative health risks (U.S. EPA 2000, 2002c; van den Berg et al. 1998).

Defined mixtures (see Supplemental Material, Appendix 1, Table 1-2; http://www.ehponline.org/docs/2007/9333/suppl.pdf) are created at a given time and place, have a reasonably defined composition, at least when emitted (before the action of chemical and biological modifications in the environment), and have components that do not necessarily possess similar properties (e.g., diesel exhaust). They are more complicated than similar mixtures but still provide a simplified conceptual construct that focuses attention on a manageable and meaningful segment of real-world environmental stres-sors. Quantitative risk assessments have been conducted for selected defined mixtures, including environmental tobacco smoke, coke oven emissions, and diesel engine exhaust (U.S. EPA 2003).

Coincidental mixtures (see Supplemental Material, Appendix 1, Table 1-3; http://www.ehponline.org/docs/2007/9333/suppl.pdf) occur by happenstance at a time or place of interest (e.g., urban air pollution). Mixture constituents do not necessarily have similar properties; the composition is not necessarily constant; and the mixture may occur frequently, occasionally, or rarely. Coincidental mixtures are the most complex of the three categories because they necessarily include all environmental stressors that are relevant for the toxicologic end point of concern. Despite the inherent complexity associated with coincidental mixtures, preliminary and screening-type cumulative risk assessments have been attempted for certain real-world situations, including urban air pollution (Caldwell et al. 1998; Fox et al. 2004; Morello-Frosch et al. 2000; Tam and Neumann 2004), consumption of home-grown vegetables by urban populations (Hough et al. 2004), and adverse impacts on specific ecosystems (Gentile et al. 2001; Obery and Landis 2002; Suter 1999; U.S. EPA 2002a, 2002d).

It is important to understand that, in contrast to similar mixtures, standard dose-addition formulae may not accurately predict the results of interactions for coincidental mixtures. Furthermore, the strength of interactive effects may differ as a function of the doses of the components depending on any nonlinearities in the dose–response relationships for the biological processes affected by the components. A further complication is that the temporal characteristics of both exposures and resulting alterations in biological processes may differ for different components of coincidental mixtures.

The three categories described above rovide a practical nomenclature for describing the types of mixtures that have been or will be the subject of cumulative risk assessments. Similar mixtures, which are defined by how they are assessed, reduce the complexity of the problem by artificially limiting the scope of the inquiry to a manageable number of stres-sors with similar characteristics. Defined mixtures, which are defined by how they are generated, are source-oriented, and lessen the difficulties by focusing exclusively on a distinct type of real-world mixture, such as emissions from certain sources or source categories. Coincidental mixtures, which are defined by the scope of the problem to be addressed, are receptor-oriented and therefore focus on people or ecosystems. They are the most complicated because they potentially include any and all relevant stressors to which people and ecosystems are exposed during day-to-day activities. It is worth noting that the the U.S. EPA guidance on risk assessment for chemical mixtures also distinguishes among mixtures that can be assessed *a*) as substances in themselves, *b*) by analogy to comparable mixtures, and *c*) as a collection of individual components (U.S. EPA 2000)

### Identifying high-priority mixtures

Priorities must be established to identify the environmental mixtures of greatest public health concern because it is impossible to characterize even a small fraction of the actual mixtures that are normally encountered during everyday life, As a first step, four attributes can be used to distinguish mixtures that deserve attention from researchers, risk assessors, and regulators: scope of exposure, nature of exposure, severity of effects, and likelihood of interactions. Based on these criteria, a high-priority mixture would have the following characteristics:

- Scope of exposure: A large number of organisms, communities, or populations are exposed to the mixture and/or a significant number of susceptible organisms, communities or populations are exposed to the mixture.
- Nature of exposure: The magnitude, duration, frequency, and/or timing of exposure to the mixture raises concerns about possible adverse effects.
- Severity of effects: The known or suspected adverse outcomes of exposure to the mixture are of a nature or consequence that suggests risks are likely to be unacceptable.
- Likelihood of interactions: Adverse effects from exposure to the mixture are not likely to be characterized adequately based on knowledge of known effects of individual mixture components acting separately.

For similar mixtures (see Supplemental Material, Appendix 1, Table 1-1; http://www.ehponline.org/docs/2007/9333/suppl.pdf), application of these criteria suggests that among the high-priority mixtures are persistent organochlorine pollutants. These include polychlorinated dioxins, polychlorinated dibenzofurans, and polychlorinated biphenyls (PCBs) (Birnbaum and DeVito 1995; Hamm et al. 2003; Walker et al. 2005); polycyclic aromatic hydrocarbons (PAHs) including benzo[*a*]pyrene, benzo[*f* ]fluoranthene, and dibenzo[*ah*]anthracene (Armstrong et al. 2004; Bostrom et al. 2002; Central Pollution Control Board 2003; Department of the Environment and Heritage 1999); organophosphate (OP) pesticides including chlorpyrifos, diazinon, azinphos methyl, and oxydemeton-methyl (Barr et al. 2004; U.S. EPA 2002c); hormonally active agents including some organohalo-gens, phthalate acid esters, bisphenol, PCBs, and phytoestrogens (Damstra et al. 2002; Safe et al. 2002); and neurotoxins including lead (Pb), mercury (Hg), organic solvents, organochlorine pesticides, and PCBs (Needham et al. 2005a; Tilson 2000).

Among defined mixtures (see Supplemental Material, Appendix 1, Table 1-2; http://www.ehponline.org/docs/2007/9333/suppl.pdf), examples of high-priorities are drinking water disinfection by-products including tri-halomethanes, haloacetic acids, and haloace-toniriles (Feron et al. 2002; Simmons et al. 2002); diesel exhaust including hundreds of chemicals in either particulate or gaseous phase (Health Effects Institute 1995; U.S. EPA 2002b); and coal-fired power plant emissions including particulate matter, heavy metals, nitrogen oxides, and sulfur dioxide (Levy et al. 2002; Sram et al. 1996).

Among the virtually infinite number of possible coincidental mixtures (see Supplemental Material, Appendix 1, Table 1-3; http://www.ehponline.org/docs/2007/9333/suppl.pdf) that occur in the real world, there are three obvious examples of high-priority exposures. First, workers in industries such as construction and underground mining are likely to be exposed to myriad hazardous agents (Tarcher 1992). Categories of potential stressors that can contribute to these complex mixtures are listed in Supplemental Material, Appendix 1, Table 1-4 (http://www.ehponline.org/docs/2007/9333/suppl.pdf), and examples of the kinds of mixtures typically encountered in various industries are provided in Supplemental Material, Appendix 1, Table 1-5 (http://www.ehponline.org/docs/2007/9333/suppl.pdf) (Tarcher 1992).

Second, in contrast to workers, who tend to be relatively young and healthy, and who spend only 40 hr per week on the job, most people, including the sick, the elderly, and the very young, typically spend a significant portion of each day indoors at home (Baker et al. 2001; Samet and Spengler 1991). As shown in the Supplemental Material, Appendix 1, Table 1-6 (http://www.ehponline.org/docs/2007/9333/suppl.pdf), there are many categories of environmental stressors commonly found inside residences, and cumulative exposures and associated risks can be unacceptably high (California Air Resources Board 2005).

Third, as illustrated in Supplemental Material, Appendix 1, Table 1-7 (http://www.ehponline.org/docs/2007/9333/suppl.pdf), many poor people, a disproportionate fraction of whom are people of color, live in blighted inner-city neighborhoods where they are exposed to a complex concoction of stres-sors. In addition, these people are also more likely to be employed in hazardous occupations, lack knowledge of environmental health issues, smoke cigarettes and drink alcohol, have a substandard diet, lack access to adequate health care, and, in general, live more stressful and less healthful lives. (Evans and Kantrowitz 2002; Evans and Marcynyszyn 2004; Gee and Payne-Sturgis 2004; Institute of Medicine 1999; O’Neill et al. 2003; Sexton 1997, 2000; Williams 1999).

## What Is the Nature and Magnitude of Cumulative Exposure?

### Concepts and definitions

**Figure 1:**
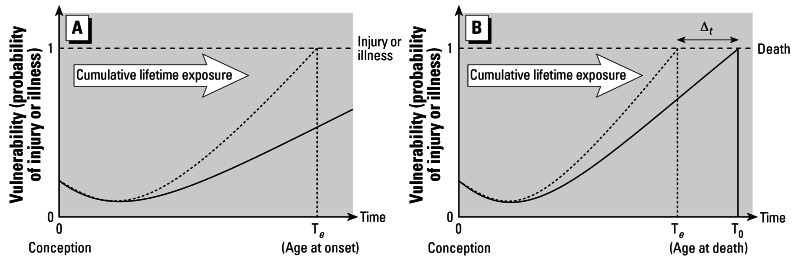
Schematic representation of possible effects from differential cumulative life-time exposure on (*A*) cumulative exposure and morbidity and (*B*) cumulative exposure and mortality [adapted from NRC (2002)]. Abbreviations: Δ*_t_*, difference in time at age of death; T*_e_*, age at onset or death for individual A; T*_e_*, age at death for individual A

Cumulative exposure refers to past and/or present exposure (including relevant background exposure) of an entity to multiple environmental stressors occurring by all pertinent routes, pathways, and sources. Exposure to mixture constituents need not be necessarily contemporaneous to produce cumulative impacts, thus it is vital to be cognizant of how the presence or persistence of multiple stressors (or their consequences) contributes to the series of biological events that cause adverse effects. Cumulative exposure assessment is the appraisal of simultaneous, overlapping, and/or sequential exposure to multiple environmental stressors that may contribute to harmful outcomes. A major motivation for conducting cumulative exposure assessments is to help determine whether differential exposure of individuals, communities, or populations to environmental mixtures causes increased vulnerability (Figure 1). Differential exposure refers to differences in the magnitude, duration, frequency, or timing of exposure as well as dissimilarities in historical and background exposure levels and related body burden that can affect the likelihood, nature, and severity of adverse effects. The term “vulnerability” is used here to mean the intrinsic propensity of an exposed entity to experience adverse effects from external agents, events, perturbations, or stresses (U.S. EPA 2003). A brief overview of important cumulative exposure issues is provided in Supplemental Material, Appendix 2 (http://www.ehponline.org/docs/2007/9333/suppl.pdf).

Cumulative exposure assessment is methodologically and computationally more complex than traditional single-chemical, single-pathway, single-source assessments because assessors must take account of *a*) temporal concordance (exposure to two or more stressors within a timeframe consistent with their toxicologic mode(s) of action); *b*) spatial concordance (contact with two or more stressors within a geographic area or physical space consistent with the possibility of cumulative exposure); and *c*) sociodemographic concordance (cumulative exposure of potentially vulnerable groups such as pregnant women, fetuses, children, the sick, the elderly, and the socioeconomically disadvantaged, which occurs across both temporal and spatial dimensions) (Mileson et al. 1999; U.S. EPA 2003).

In practice, measuring or estimating concurrent exposure to multiple stressors is not straightforward, even if the toxicologically relevant temporal, spatial, and sociodemographic aspects are known. For example, cumulative exposure assessment necessitates assessment of background exposure. Although the term “background exposure” has been used to describe a variety of conditions and situations, we use it here to mean the combined exposure to toxicologically relevant environmental stres-sors that are not necessarily the focus of the assessment but that may contribute to the cumulative risks being considered. Under this definition, both naturally occurring (e.g., terpenes emitted by pine trees) and artificially created (e.g., xylenes emitted by both mobile and stationary anthropogenic sources) compounds can be included, their influence may be localized or widespread in the environment, and their sources may or may not be known. The important point is that background exposures, which can occur across all exposure sources, pathways, and routes, must be evaluated as an intrinsic part of cumulative exposure assessment, if for no other reason than to exclude them as a significant contributor to the risks being assessed.

In addition to background exposures, it is usually also necessary to determine historical exposures as well. Determining the exposure history for the period of interest often means that we are concerned about exposures that occurred 10 or more years ago. This is a difficult challenge for exposure assessors because relatively scant exposure-related information is available for most real-world mixtures of public health concern. In some cases, past exposure (over months or years) to persistent compounds can be estimated from concentrations of the chemicals, their metabolites, or reaction products in bodily tissues or fluids (e.g., Pb in blood). Other nonpersistent compounds (e.g., benzene), however, may produce adverse effects that occur long after the chemicals are no longer present in the body, although some of these chemicals do produce DNA adducts or protein adducts that can be used as exposure- and/or early-effect bio-markers (Sexton et al. 2004a). In general, accurate assessment of historical exposure to mixture constituents over the past several years, let alone the past several decades, is problematic. It is important to remember that exposures need not have actual temporal overlap in order to create interactions by changing the vulnerability of sensitive receptors.

Because the monitoring data necessary for retrospective exposure assessment are lacking in most cases, assessors typically must rely on less exact methods to estimate historical exposure, such as interviews, questionnaires, documentation of occupational histories, centralized monitoring data for air and water pollutants, or construction of exposure scenarios (NRC 1991; Sexton et al. 1995b, 2004a). However, ongoing advances in technology, data collection methods, and scientific understanding hold out the promise of more accurate assessments in the future.

### Six trends benefiting cumulative exposure assessment

There are currently at least six trends that suggest cumulative exposure assessment may, over time, become easier and more straightforward. First, there are ongoing efforts to establish state and federal environmental health tracking systems that provide for the systematic collection, integration, analysis, interpretation, and dissemination of information about environmental hazards, including sources, environmental concentrations, exposures, doses, and potentially related health effects. The creation of linked monitoring systems, databases, and registries offers the prospect of better data on cumulative exposures and improved understanding of the connection between combined exposures and chronic diseases such as diabetes, arteriosclerosis, and cancer (Litt et al. 2004; McGeehin et al. 2004).

Second, large-scale prospective studies, such as the National Children’s Study (NCS) (if eventually funded), will provide data on exposure to multiple environmental agents during various stages of the life cycle. The NCS is planned to be a national longitudinal study of environmental influences, including physical, chemical, biological, and psychologic stressors, on children’s health and development from conception to early adulthood. Multiple exposure measures will be collected on a cohort of 100,000 children, and the results will enable risk assessors to more accurately assess children’s cumulative exposure to a broad range of environmental stressors (Barr et al. 2005; Needham et al. 2005b).

Third, the growing availability of specific and sensitive biologic markers of exposure, effects, and susceptibility provides an increasingly effective means of assessing cumulative exposure and, potentially, associated health effects (Needham and Sexton 2000; Sexton 2006; Sexton et al. 2004a, 2004b, 2005, 2006; Weis et al. 2005). A case in point is the “National Report on Human Exposure to Environmental Chemicals” (the National Report), which is published periodically by the Centers for Disease Control and Prevention (2005). The National Report provides reference ranges for exposure to multiple chemicals among the U.S. population, subdivided by age, sex, and ethnicity. The first report was published in March 2001, the second in January 2003, and the third, which presents exposure data for 148 chemicals, in July 2005. Collectively, these data provide an indication of the range of nonoccupational exposure for both individual chemicals (e.g., Pb, Hg) and similar mixtures of chemicals (e.g., organochlorine-based pesticides, organophosphate-based pesticides, carbamate-based pesticides, PCBs, dioxins, furans, phthalates, phytoestrogens). Although all chemicals and chemical classes are not measured in the same individuals, and data for children younger than 12 years are only available for a few chemicals such as lead and mercury, the National Report provides an invaluable resource for approximating distributions of body burden levels, identifying high-priority exposures, and understanding how levels of multiple chemicals are changing over time.

Fourth, advances in biomedical sciences including ongoing developments in genomics, proteomics, metabolomics, transcriptomics, nanotechnology, and medical imaging promise to eventually revolutionize the assessment of cumulative exposure and related health risks. These methods provide new quantitative tools for assessing biological response to cumulative environmental exposure, thereby affording expanding opportunities to gain an in-depth understanding of exposure-related events that occur along the pathway from human contact with environmental mixtures to eventual environmentally-induced discomfort, dysfunction, disability, disease, and death. For example, the National Institute of Environmental Health Sciences (Research Triangle Park, North Carolina) is currently developing an “exposure biology” initiative aimed at advancing knowledge of gene–environment interactions in model disease processes so that we can attain the same level of individual-level precision as is being achieved through the sequencing of the human genome (Schwartz et al. 2005).

Fifth, a variety of innovative technologies, including improved environmental sensors (Weis et al. 2005), geographic information systems (Meliker et al. 2005; Weis et al. 2005), and Bayesian statistical techniques (Burstyn and Kromhout 2002; Ramachandran 2001; Ramachandran and Vincent 1999), offer the possibility of enhanced retrospective assessment of historical exposure. For example, microscale sensors, such as tiny, inexpensive, personal exposure monitors that use fluorescence or cell function to detect chemicals in nanoscale sample volumes, and macroscale sensors, such as infrared radiation–based monitors that detect sulfur and nitrogen oxides in industrial-stack effluents, are making it easier and less expensive to measure actual exposures as well as environmental concentrations (Weis et al. 2005). Spatiotemporal visualization tools have been used to produce smooth, continuous space–time maps that assign exposure estimates through a spatial query procedure, as well as smooth, continuous temporally variant histograms and scatter plots that allow for examination of relationships between exposure-related variables at any moment in time (Meliker et al. 2005). Bayesian statistical approaches, which take prior information into account in determination of probabilities, have been used to assess occupational exposure retrospectively as a function of space and time through systematic synthesis of a combination of expert judgments, exposure models, historical data about workplace conditions, and available exposure measurements (Burstyn and Kromhout 2002; Ramachandran 2001; Ramachandran and Vincent 1999).

Sixth, numerous computer models have been developed by federal agencies, academic researchers, and private-sector scientists to simulate longitudinal exposures to multiple chemicals via multiple pathways from multiple sources. These ongoing efforts have necessarily shifted the focus from traditional, source-oriented approaches to receptor-oriented approaches (Price and Chaisson 2005). Currently, there are several PC-based cumulative exposure models that can be used to calculate exposure histories for chemical mixtures, including the Lifeline, Calendex, CARES (Cumulative and Aggregate Risk Evaluation System), SHEDS (Stochastic Human Exposure and Dose Simulation), and APEX (Air Pollutants Exposure) models (Price and Chaisson 2005). As more data become available and understanding of cumulative exposure improves, the accuracy of results will get better and the use of these and subsequent models will increase.

## What is the Mechanism and Consequence of Interactive Effects?

In addition to cumulative exposure evaluation, understanding mechanisms of interaction among mixture constituents is important for realistic risk assessment because mechanistic knowledge allows for quantitative predictions of the nature and consequences of co-exposure to different stressors. Morevoer, mechanistic framing of relevant questions is more productive for research planning than simple dose-addition or response-addition formulae.

### Types of interactive effects

There are three general types of interactions among mixture components that can affect toxicologic response to the whole mixture (Thomas et al. 2002; U.S. EPA 2000):

- Agent-to-agent interactions prior to crossing the boundaries of an organism can occur among mixture constituents, such as between airborne hydrocarbons and NO_x_ (nitrogen oxide) in the presence of ultraviolet radiation to produce tropospheric ozone. These sorts of interactions are not discussed in this article.
- Toxicokinetic interactions, which occur once mixture constituents have crossed an organism’s boundaries, can take the form of enhancement or inhibition of absorption, distribution, metabolism, and elimination of one or more mixture components.
- Toxicodynamic interactions as a result of exposure to mixture constituents, their metabolites, or reaction products can alter mechanisms of damage, repair, compensation and signaling.

The effect of interactions (or lack thereof) among mixture components on empirically observable dose–response relationships for toxicity can be divided into four broad categories: independence, dose additivity, synergism, and antagonism (Hertzberg and Teuschler 2002; U.S. EPA 2003). If agents in the mixture act independently (which is to say that they are unconnected in any way), then the mixture toxicity is qualitatively and quantitatively equivalent to their separate distinct effects—essentially “response additivity.” If the mixture constituents do not act independently (e.g., they have a similar mechanism of toxic action) but no significant interactions occur, then the toxicologically relevant dose is considered to be equivalent to the sum of individual constituent doses. This situation is referred to as “dose addition” or “additive dose.” When the toxic effect of the mixture is greater than that expected for the sum of individual constituent doses, which is that effects of combined doses are more-than-additive, the interactions are said to be synergistic. Conversely, when the toxic effect of the mixture is less than that expected under the dose additivity assumption, the interactions are said to be antagonistic (Hertzberg and Teuschler 2002). In the subsequent discussion we focus on identifying instances where synergism or antagonism may occur, and describing the responsible interaction mechanisms.

### Toxicokinetic interactions

Toxicokinetic interactions occur when one factor affects the transport or metabolism of an environmental chemical in a way that changes the concentration and time profile of the ultimate active metabolite at the internal body site of action for causing some adverse effect. Examples of this include inhibition or induction of active transport or metabolizing systems. Physiologically based pharmacokinetic (PBPK) models, which are the basis for mathematical analysis of pharmacokinetic interactions, are discussed in the Supplemental Material, Appendix 3 (http://www.ehponline.org/docs/2007/9333/suppl.pdf). Selected examples of toxicokinetic interactions are discussed below.

#### Enzyme and active transport induction

The major ways that exposure to one chemical can change the metabolism of another chemical follow from the simple molecular picture underlying Michaelis-Menten kinetics (See Supplemental Material, Appendix 3; http://www.ehponline.org/docs/2007/9333/suppl.pdf). If exposure to one substance (e.g., some PCBs) causes enhanced transcription and translation of a cytochrome P450 (CYP) enzyme that metabolizes another [e.g., the induction of CYP2E1 by ethanol (Ingleman-Sundberg et al. 1994) or nicotine (Micu et al. 2003)], one would expect an increase in *V*_max_ (maximum rate of production of metabolic product) for the second chemical. This would decrease the local and systemic availability of the parent (substrate) chemical and increase the rate of production of an active metabolite of the parent chemical, if the active metabo-lite results from metabolism by the induced CYP enzyme.

#### Competitive inhibition and other variants

If two substrates bind to the same active site on the same enzyme so that binding of one substrate prevents the binding of the other, then the two substrates are said to be “competitive” inhibitors. In the context of the Michaelis-Menten enzyme kinetic equation, the effect of this competition is to increase the *K_m_* (the Michaelis constant, defined as the substate concentration that elicits half the maximum rate of production of metabolic product) by an amount that depends on the relative binding affinity to the active site and the concentration of the inhibiting/competing substrate. In addition to competitive inhibition, there are at least two other modes of interaction—“uncompetitive” and “noncom-petitive”—that have been described within the classic Michaelis-Menten framework (see Supplemental Material, Appendix 4; http://www.ehponline.org/docs/2007/9333/suppl.pdf).

#### Modification of uptake and local elimination

Sometimes the cumulative changes caused by one toxicant have implications for internal exposure to another toxicant. To illustrate, particles are cleared from the lung in two phases with very different half-lives, depending on the site of deposition. Particles deposited on the ciliated tracheobronchial epithelium are swept up the bronchial tree out of the lung and swallowed with half-lives on the order of 3 hr in never-smokers (Möller et al. 2004). By contrast, particles deposited in the deep lung (alveoli and terminal respiratory airways) must be cleared by processes involving macrophages, with half-lives of 100 days or more in nonsmokers. In smokers, however, clearance times increase linearly with the cumulative number of pack-years of cigarette smoking (Bohning et al. 1982; Möller et al. 2001). Such reductions in long-term clearance are likely to increase the chronic accumulation of small particles and enhance associated effects (Hattis and Silver 1994).

### Toxicodynamic interactions

Toxico-dynamic interactions are generally less understood than toxicokinetic interactions. A systematic way to approach this subject is in terms of levels of biological organization:

- Subcellular phosphorylation cascade (“second messenger”) signaling processes operating on a time scale of seconds to minutes.
- Cellular processes involving gene transcription and translation, and most genomic defense mechanisms (DNA repair, apopto-sis, cell cycle arrest), typically operating on a time scale of minutes to hours.
- Cell-neighbor (“juxtracrine”) communication processes (contact inhibition and other signaling among adjacent cells via “gap junctions”).
- Organ- and tissue-level control and feedback processes influencing the choices of individual cells among symmetrical and asymmetrical proliferation of stem cells, differentiation, long term survival and functioning without proliferation for end-state differentiated cells, and apoptosis.
- Local functional control to support short-term tissue needs (e.g., dilation of blood vessels in response to a local drop in oxygen tension, perhaps from local muscle activity).
- Intersystem feedback controls via neural and endocrine signaling.

In the discussion below we use this classifica-tion scheme to provide some examples of tox-icodynamic interactions.

#### Subcellular phosphorylation cascade signaling processes

Cells have elaborate internal signaling systems that mediate rapid (seconds to minutes) changes as diverse as choices to respond to DNA damage in various ways, initiate cell division, or differentiate to express the end state functions needed in a particular tissue. Many of these signals involve transferring phosphate groups to alcoholic amino acids (e.g. tyrosine, serine) in specific proteins. For example, double-strand DNA breaks produced by ionizing radiation are initially sensed and communicated via the phos-phorylation of the ATM protein (ATM is “mutated in ataxia-telangiectasia,” a genetic disease characterized by unusual sensitivity to ionizing radiation, among other effects).

#### Gene transcription/translation and genomic defense mechanisms

An array of cellular defenses is induced in response to genetic damage, thereby creating an opportunity for exposure to one chemical to affect vulnerability to another chemical (Bartek and Lukas 2001). Gene transcription and translation responses typically occur on a time scale of minutes to several hours. The end results of these processes include cell cycle arrest, either at the boundaries between G_1_ and S where DNA begins to be synthesized, or in G_2_ after DNA is synthesized but before the cell is committed to the chromosome separation process of mitosis. Cell cycle arrest (Shackelford et al. 1999) can be beneficial in allowing more time for DNA repair before replication-related “fix-ation” of DNA lesions into essentially permanent changes in the information coded in DNA (mutations).

Alternatively, DNA repair can occur by several different processes (Hoeijmakers 2001) including one (methyl–guanine–methyl trans-ferase) that involves apparently permanent inactivation of a particular protein that serves as both an enzyme and the acceptor for the removed methyl group. Regeneration of the active form of this enzyme through new synthesis occurs over a period of days, implying that cells exposed to one alkylating agent may well be more sensitive to the action of another alkylating agent during that interval (Gerson 1988). Apoptosis—or programmed cell suicide—is a backstop mechanism that appears to be particularly prominent as a response in stem cells capable of indefinite proliferation. Alternatively, DNA damage can induce some cells to enter a senescent state in which they are permanently withdrawn from the cell cycle, preventing possible further mutation to cancer in that cell but preserving, for a while, some level of differentiated function (Campisi 2005).

#### Changes in signaling among adjacent cells via gap junctions

A good example is the interference with the cell communication via transfer of small molecules across gap junctions involving connexin proteins. Such inhibition is thought to be an important mechanism of promotion of cancer cells, releasing the initiated cells from growth inhibition by untransformed neighboring cells (Mesnil 2002; Trosko et al. 2004). It is plausible that chemicals acting by this mechanism may have synergistic interactions with chemicals that act by primary genetic mechanisms to produce cancer. The chlorinated insecticides lindane and chlordane are prime examples of such inhibitors (Caruso et al. 2005).

#### Organ and tissue-level control and feedback processes

Organ- and tissue-level feedback can influence the choices of stem cells among symmetrical and asymmetrical proliferation. Epithelia and underlying mes-enchymal-derived tissues are in constant dialogue that maintains desirable numbers of cells at all stages in differentiation, from the earliest stem cells through rapidly proliferating progenitor cells to terminally differentiated functional cells. These processes have been studied most intensely in intestinal systems, where a cell’s position in the structure of the proliferating crypt and functional villus provides information on each cell’s position in the stem-to-differentiated cell sequence (Sancho et al. 2004). Among the mediators of tissue responses to radiation and probably other sources of oxidative stress is transforming growth factor beta (TGF-β). This normally resides outside of cells in close association with a specific inhibitor. The inhibitor is released on exposure of intestinal tissue to radiation and likely to other oxidative stimuli, leading to increased apoptosis and probably other defensive responses (Thavaraj et al. 2005). TGF-β also regulates growth and apoptosis in other contexts (Arsura et al. 2003), including development (Tsutsumi et al. 2002).

#### Changes in cell replication

Enhancement of cell replication may be one of the more common sources of extreme high-dose nonlinearities in chronic animal testing and, when present in humans due to an interacting factor (e.g., viral infections), could be a source of synergistic interactions in people. For example, it is well documented that there is a synergistic interaction between aflatoxin exposure and hepatitis B infection in the induction of liver cancer (Kew 2003). The precise mechanism has not been elucidated, but it is likely that hepatitis B infection induces increased cell replication, making the cells more vulnerable to mutation by aflatoxin B partly because of reduced time for DNA repair.

#### Neural signaling

Neurons communicate by secreting transmitter substances across small spaces between adjacent cells. Some environmental toxicants such as organophosphate and carbamate pesticides, as well as many important drugs, act by affecting transmitter-mediated communication in one way or another. The organophosphate and carbamates are anti-cholinesterase agents that act by binding to the active site of acetylcholinesterase and inhibiting its action, leading to greater persistence of the acetylcholine, and hence longer transmission of the signal.

#### Endocrine and hormonal signaling

Continuing stimulation of an endocrine pathway with an agonist (a drug or environmental chemical that binds to a receptor and stimulates it to induce a signaling cascade similar to the natural ligand) will tend to induce some offsetting desensitization of the system. This can take the form of a reduction in the number of the excessively stimulated receptors (down-regulation), which reduces the response of the system to the natural ligand (or other external agonists) or other mechanisms of desensitization such as down-regulation of downstream steps in the biological response (such as altering the strength of the phosphorylation or second-messenger cascade that normally follows receptor binding).

#### Autoimmune responses and other learned responses

Immune recognition mechanisms rely on co-stimulation of ultimate effector cells by both specific antigens and less specific chemical mediators, which signal the presence of nonspecific damage by an invading organism. Even exposure to relatively chemically inert environmental pollutants such as silica dust or to chemicals that are only slowly metabolized to potentially reactive agents such as hexachlorobenzene can affect the immune response, which in turn can increase vulnerability to other environmental exposures (Pieters et al. 2003). See Supplemental Material, Appendix 5 (http://www.ehponline.org/docs/2007/9333/suppl.pdf), for a brief discussion of immune-mediated drug-induced liver diseases.

### Developing better understanding of combined effects

Development of a more comprehensive picture of vulnerability changes in response to cumulative exposure to environmental mixtures will depend on enhanced quantitative understanding of mechanisms that underpin the system of feedback processes controlling vital systems in humans and other organisms. For long-term interactions, it is particularly important to understand potential vulnerabilities in the systems by which our homeostatic “set points’ are themselves “set” during development, then maintained through growth maturation and adulthood, and finally start to break down with advancing age. Set-point systems necessarily involve three components: sensors that monitor the levels of key parameters; signaling mechanisms that communicate needs for adaptive responses; and effector mechanisms that take action to offset detected departures from established set points. These set-point systems must necessarily operate on different scales of biological organization, time, and physical distance.

#### Biological organization

There are at least four distinct levels of biological organization: *a*) the subcellular level, which, for example, controls activation versus silencing of portions of the genome (apparently subject to disruption in very early embryos prior to organo-genesis via exposures to agents known for their DNA-damaging properties; Kimmel et al. 1993); *b*) the cellular level, where there must be controls on pool sizes (concentrations) and turnover rates of key metabolic intermediates, and the adenosine triphosphate energy currency of the cell; *c*) the tissue/organ level, where similar and different cell types coordinate their activities to perform physiological functions at required rates, such as the control of blood electrolyte levels by the kidney, the orderly sequential contraction of muscle cells in different parts of the heart, and the coordinated processing of information from the retina to form and identify an image of an object by the brain; *d*) the system/organism level, where the activities of multiple systems must be coordinated to accomplish centrally-directed tasks (e.g., the regulation of heart rate, breathing rate, dilation of numerous blood vessels, and muscle activity needed to play soccer).

#### Time

Biological systems are known to undergo regular adaptive fluctuations in time, with some changes tied to external events (e.g., circadian rhythms coinciding with light–dark cycles) and others to internal events (e.g., cell cycle, menstrual cycle). Much attention has been focused recently on understanding the daily, weekly, and yearly cycles in acute coronary events, which can be modi-fiable by aspirin and beta blockers, and giving hope that causal mechanisms can eventually be determined (Strike and Steptoe 2003).

#### Physical distances

Some signaling and feedback systems must operate on a diversity of scales, including: micron or submicron scales (e.g., coordinated transport of chromosomes to the centrioles during mitosis); between adjacent cells (e.g., via gap junction communication, across synaptic gaps between neurons, or between neurons and muscle cells); over larger distances via concentration gradients of signaling molecules that are essential for segmentation of the embryo, establishment of the anterior/posterior ground plan, distinction between dorsal versus ventral sides of the embryo, and migration of future neurons to appropriate locations for differentiation and synaptogenesis; and, finally, over centimeters and even meters via specific nervous signals and endocrine hormones circulating in the blood.

Development of homeostatic controls is likely to occur first at subcellular levels of biological organization, operate over short distances, and respond with relatively rapid time scales. As development progresses, layers of control are added in sequence from local (within organs/tissues, such as the cardiac pacemaker) to distant (e.g., neural and chemical signals) (Adolf 1968) and, correspondingly, from shorter to longer response times. At present, we have inadequate quantitative understanding of how controls at these different levels work, and only an elemental sense of the initial set up. In short, we need to learn how the set points are actually set and maintained. Furthermore, we need to understand how programming occurs for allostatic adaptations (Schulkin 2003), which allow the system to adjust set points in anticipation of changing conditions (e.g., salivation in anticipation of a meal).

To better understand these biological phenomenon, we need to expand research, especially in the field of systems biology (Cho and Wolkenhauer 2003; Kim and Tidor 2003; Levchenko 2003; Zhu et al. 2004), and develop and apply new ‘bridging’ biomarkers of effect that can relate biological consequences across species (MacGregor 2003). As new methods and tools become available, risk analysis of and time-related adverse effects will evolve beyond current rudimentary approaches, such as modifying data on no observed adverse effect level/lowest observed adverse effect level using uncertainty factors, and the more recent benchmark dose methods (Allen et al. 1994). A brief summary of currently available methods for assessing cumulative health risk from chemical mixtures, along with comments on their strengths and weaknesses, is provided in Supplemental Material, Appendix 6 (http://www.ehponline.org/docs/2007/9333/suppl.pdf).

## Conclusions

Cumulative risk assessment is currently hampered by three interrelated problems: *a*) Relatively little is known about the magnitude, duration, frequency, and timing of cumulative exposure to important environmental mixtures. *b*) Scant evidence is available on whether mixture-related effects are antagonistic, synergistic, or additive at exposure levels typically encountered by people. *c*) There is inadequate knowledge and insufficient understanding about interactive mechanisms of toxicity that occur among mixture constituents. In the near-term, quantitative assessment of cumulative risks depends not only on targeted research but also on development of science-based methods and procedures for using existing exposure and effects data to characterize mixture-related health risks with an acceptable degree of precision. This will unavoidably involve science policy decisions about how best to bridge the gap between the scarcity of hard scientific evidence and the need to estimate cumulative risks as an integral part of risk management decisions.

Cumulative risk assessment will be most useful to decision makers when it can help answer a fundamental question: “Do the uncertainty/safety factors built into the conventional risk assessment process adequately protect public health and ecologic resources from cumulative effects with a sufficient margin of safety?” To be relevant, therefore, cumulative risk assessment must provide guidance about which, if any, of the innumerable environmental mixtures that are part of our day-to-day lives represent important health risks, where “important” means there is a reasonable likelihood that combined effects of mixture constituents at realistic exposure levels constitute a serious health risk that is not adequately accounted for by traditional risk assessment methods.
